# A multilingual dataset of COVID-19 vaccination attitudes on Twitter

**DOI:** 10.1016/j.dib.2022.108503

**Published:** 2022-08-02

**Authors:** Ninghan Chen, Xihui Chen, Jun Pang

**Affiliations:** a Faculty of Science, Technology and Medicine, University of Luxembourg, Esch-sur-Alzette L-4364, Luxembourg; b Interdisciplinary Centre for Security, Reliability and Trust, University of Luxembourg, Esch-sur-Alzette L-4364, Luxembourg

**Keywords:** COVID, Vaccination, Vaccine hesitancy, Twitter, Dataset, Health

## Abstract

Vaccine hesitancy is considered as one main cause of the stagnant uptake ratio of COVID-19 vaccines in Europe and the US where vaccines are sufficiently supplied. A fast and accurate grasp of public attitudes toward vaccination is critical to addressing vaccine hesitancy, and social media platforms have proved to be an effective source of public opinions. In this paper, we describe the collection and release of a dataset of tweets related to COVID-19 vaccines. This dataset consists of the IDs of 2,198,090 tweets collected from Western Europe, 17,934 of which are annotated with the originators’ vaccination stances. Our annotation will facilitate using and developing data-driven models to extract vaccination attitudes from social media posts and thus further confirm the power of social media in public health surveillance. To lay the groundwork for future research, we not only perform statistical analysis and visualization of our dataset, but also evaluate and compare the performance of established text-based benchmarks in vaccination stance extraction. We demonstrate one potential use of our data in practice in tracking the temporal changes in public COVID-19 vaccination attitudes.

## Specifications Table

**Table:** 

Subject	Social science, Data Science, Computer Science
Specific subject area	Vaccination attitudes, Sentiment analysis, Social media
Type of data	table
How the data were acquired	The data are collected through the official Twitter APIs based on effective keywords commonly used in previous collections. The annotation is conducted by a group of 10 multilingual volunteers in three rounds.
Data format	Raw (Primary)Labeled (Secondary)
Description of data collection	The data contains the IDs of 2,198,090 tweets related to COVID-19 vaccinations from four countries in Western Europe which span about 14 months. The tweets are filtered according to keywords that are commonly adopted in previous vaccine-related data collections. To enable data-driven methods to extract public attitudes, 17,934 tweets are annotated with affective vaccination stances by 10 multilingual students among which a high-level agreement is achieved.
Data source location	*Country:* France, Germany, Belgium, Luxembourg
Data accessibility	Repository name: Zenodo Data identification number: 10.5281/zenodo.5851407 Direct URL to data: https://doi.org/10.5281/zenodo.5851407
Related research article	N.A.

## Value of the Data

• Our dataset contains the IDs of 2,198,090 tweets relevant to the discourses on COVID-19 vaccination for about 14 months after the onset of the pandemic. Its large scale and long-time span allow researchers to study the vaccination attitude evolution in Western Europe before and after the first COVID-19 vaccine was approved.
• The over 17,000 tweets annotated with vaccination attitudes facilitate developing and validating new data-driven methods, e.g., in Natural Language Processing (NLP), to extract vaccination attitudes and other subjective opinions from social media posts.
• The validated performance of existing text-based NLP methods for opinion extraction demonstrates the power of social media in tracking fine-grained temporal changes in vaccination attitudes on a daily or weekly basis. The tracking can subsequently lead to timely and proactive interventions.
• The multilingualism of our annotated tweets provides a reliable data source to evaluate existing and new language transformers in dealing with multilingualism.

## 1 Data Description

We released the IDs of 2,198,090 tweets related to COVID-19 vaccines from 54,381 active Twitter users between January 20, 2020 and March 15, 2021, spanning about 14 months. The tweets originate from users located in four adjacent Western European countries: Belgium, Germany, France and Luxembourg. One important reason for our selection is that they can well portray the first group of COVID-19 vaccine receivers and other European countries hit badly by the pandemic. We manually annotated 17,934 tweets with affective vaccination stances (i.e., positive, negative and neutral). Due to IRB review and the Twitter Terms of Service, only tweet IDs are published. All tweets are publicly accessible and researchers are recommended to download them with official Twitter APIs.

Although social media posts have been used to study vaccination attitudes since the outbreak of the pandemic [1], only a few datasets are publicly available. Pierri et al. [2] published a dataset collected from Twitter and Facebook recording Italian users’ discussions about vaccination. DeVerna et al. [3] released the CoVaxxy dataset composed of English-language tweets about the COVID-19 vaccination generated from the US. Chen et al. [4] published the MMCoVaR dataset which contains only 24,184 tweets related to COVID-19 vaccines, spanning less than one month. Our dataset differs from the above datasets in three aspects. First, our released tweets cover a sufficiently long period before and after the administration of the first COVID-19 vaccine. This enables studies on the evolution of public vaccination attitudes. Second, the tweets are from four countries with different official languages. This feature enables our dataset to facilitate developing natural language processing methods dealing with multilingual texts. Last but not least, our annotated tweets allow for utilizing established data-driven methods to learn vaccination attitudes from tweets, and facilitate the development of new methods.

Our release consists of two files: all tweets and annotated tweets. The former lists the IDs of the 2,198,090 collected tweets while the latter is composed of a table of 17,934 rows with two columns: tweet id and label. Each row corresponds to a tweet identified with the tweet id and the label field gives its affective attitude towards vaccination. Specifically, we have five labels which are described as follows:

• **Positive (PO):** The originator expresses his/her support for the vaccines or vaccination in the sense that the vaccines or vaccination can effectively protect the public, and will be or has been vaccinated.
• **Negative (NG)**: The originator expresses doubts or disbelief about the effectiveness of the vaccines or vaccination in combating the pandemic, or hesitates or refuses to be vaccinated.
• **Neutral (NE)**: No explicit attitude or intention is expressed.
• **Positive but dissatisfaction (PD)**: The originator expresses dissatisfaction or complaints about the current policies or measures against COVID-19, but still holds a positive attitude towards vaccination.
• **Off-topic (OT)**: The content is irrelevant to COVID-19 vaccines or vaccination.

We give some examples for each label in Table 1. Special attention should be paid to the label PD. We notice that there exist a large portion of tweets expressing the originators’ negative feelings or disagreement about the way the governments handled the pandemic such as complaints about lock-downs. However, the originators still showed their belief in vaccines as an effective and ultimate measure to beat the virus. Such tweets use terms which are negative inherently and if not explicitly separated, they will confuse classification methods with the ones that should be labeled as NG.

**Table 1:** Tweet examples.

Label	Example (Translated to English)
**PO**	We have a new weapon against the virus: the vaccine. Hold together, again.
**NG**	My daughter, a nurse at the AP-HP, on the vaccine ”Ah ah ah! They don't evendream about it, they start with the old ones so that we can attribute the side effects to age”.
**PD**	It's bad enough for individuals to refuse #COVID19 #vaccines for themselves.But forcing a mass vax site to shutdown, knowing it means vaccines may go to waste, is criminal. Call it pandemicide.
**NE**	Have any diabetics been vaccinated? I need some information
**OT**	a 10% discount on pet vaccinations next week.

**Fig. 1:**
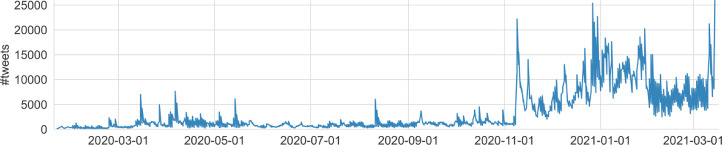
The temporal distribution of tweets on a daily basis. Each point represents the number of collected tweets posted in one day between 1/20/2020 and 3/15/2021. The fluctuations indicate the changes in the popularity of COVID-19 vaccine-related topics.

**Fig. 2:**
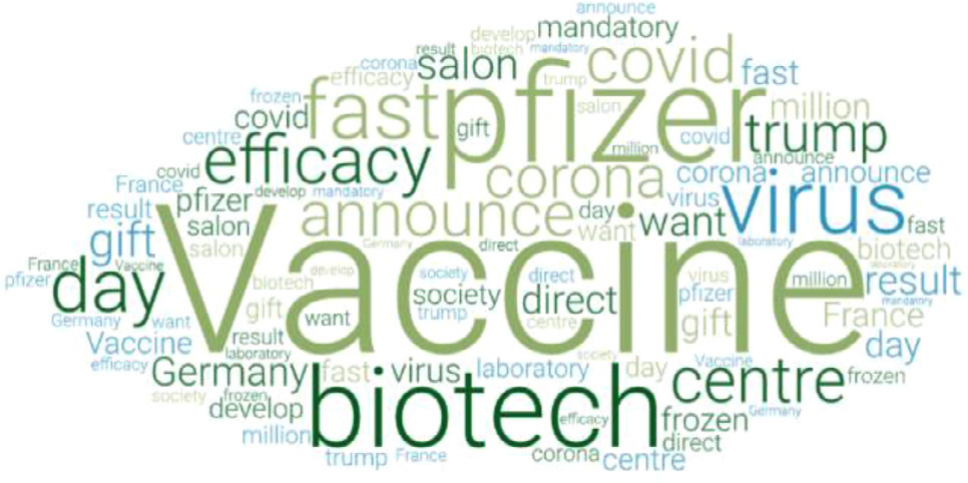
Word cloud of the tweets posted between 11/9/2020 and 11/11/2020. The size of a word implies the relative frequency of its occurrences in the period. All tweets are translated into English before the calculation of frequent words.

In Fig. 1, we display the number of collected tweets on a daily basis. Sudden surges of daily posts can help understand the changes of public attention and investigate possible events that cause the changes. We can see that COVID-19 vaccines or vaccination were rarely discussed before November 9, 2020, when a sudden surge occurred. Since then, the discussion around them remains popular. To understand the events that promoted the increase, we extracted the tweets that were generated in the 3 days after November 9, 2020 and created a word cloud with Natural Language Toolkit (NLTK)^1^ to identify the frequently used words (see Fig. 2). Note that all extracted tweets are first translated into English with Google translate API. The color does not have specific meanings and the font sizes illustrate the relative popularity of the words. With the highlighted words, we check the news and confirmed that Pfizer announced the 90% efficacy of its vaccine co-developed with Biotech.^2^ With a similar approach, we checked the peak on December 27, 2021 and the sudden increase on March 15, 2021. We discovered that the former tweet peak is attributed to the start of the EU Mass Vaccination Campaign while the latter increase is likely to be caused by the news that France and Germany joined other European countries to temporarily halt the use of the Oxford-AstraZeneca vaccine.

**Fig. 3:**
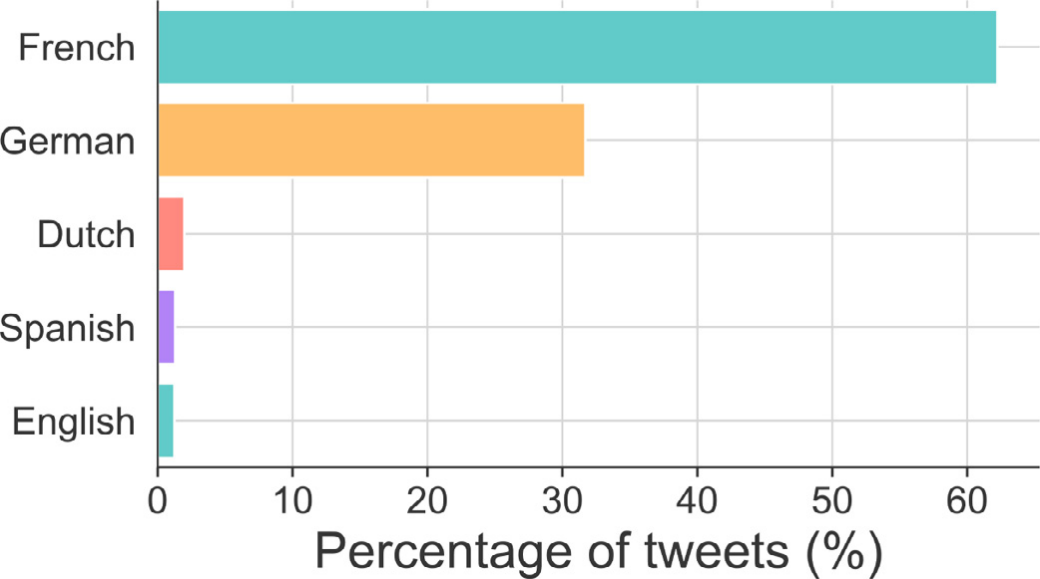
Distribution of tweets over languages. Each column represents the percentage of tweets written in a specific language. French as the official languages of three selected countries is dominant over others.

**Fig. 4:**
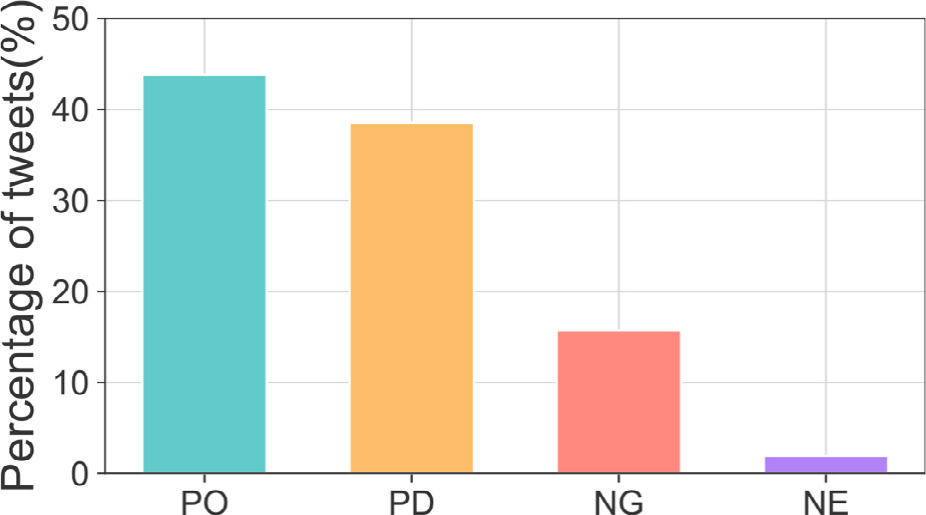
Distribution of annotated attitudes. Each column represents the percentage of tweets labeled with a specific label in our annotation dataset.

A feature of our tweet dataset is the multilingualism which is inherent in Europe. In Fig. 3, we show the distribution of the tweets in the top 5 languages which are most frequently used in our dataset. As the official language of France, Belgium and Luxembourg, French is the dominant language which is used in more than 60% of the collected tweets. Multilingualism is considered as a challenge in NLP to extract subjective opinions from texts. Researchers will benefit from our tweet dataset and our annotation in developing and validating new NLP methods to address this challenge. We depict the distribution of annotated tweets over the vaccination attitude labels in Fig. 4. Adding up those labeled both PO and PD, we can see that more than 60% of the tweets express a positive attitude toward vaccination while about 20% are associated with negative attitudes.

## 2 Experimental Design, Materials and Methods

### 2.1 Data Collection

Our data collection process comprises two steps. In the first step, we constructed the set of active users from our targeted regions, i.e., Belgium, Germany, France, and Luxembourg. We say a user is active if he/she frequently participates in vaccine-related discourse and interacts with others. We focused on these users because active users are more likely to express their true opinions through tweets. In the second step, we streamed tweets from the identified active users using manually chosen keywords.

#### 2.1.1 Active User Identification

According to the Twitter policy, the only way to get Twitter users is from the meta-information associated with tweets. Instead of direct searching tweets, we refer to a publicly available tweet dataset [5]. This continuously updated dataset contains IDs of tweets related to COVID-19 and originated from users all over the world. Our data collection spans from January 22, 2020 to March 15, 2021, and covers a period from the early stage of the pandemic to the beginning of the vaccination campaign. We downloaded the tweet IDs from the dataset which fall in this period, and then crawled the corresponding tweets with the Twitter API. In total, we downloaded 51,966,639 tweets from which 15,551,266 Twitter users are obtained. To retain users that are active and located in the targeted countries, we conducted two sequential filtering operations: *location-based filtering* and *activity-based filtering.*

*Location-based filtering*. We made use of the geographic information in the metadata of tweets to learn users’ originating countries. If a user has multiple tweets generated from different countries, although this rarely occurs among our collected users, we use the country reported in the earliest tweet. The metadata of a tweet has two fields to store the originator's locations: Geo and Place. The Geo field records the location generated by the user's device while the Place field stores the geographic information provided by users. The Geo information is accurate and in a unified format that can be automatically parsed. However, only about 2% of tweets come with Geo values. As a result, we use the Place field which is usually ambiguous when the Geo field is not available. To regularize the location format and remove the ambiguity, we leveraged the ArcGis Geocoding, which is widely used in previous research for the same purpose [6]. For example, the Place field value Moselle, input by a user, is converted into a machine-resolvable location including city, state and country: Mosselle, Lorraine, France. Among the selected users, more than 70% have at least one geographical field filled. We removed the users located outside the four countries and finally obtained 767,583 users.

*Activity-based filtering****.*** We built a user interaction graph to screen out inactive users. A user interaction graph is undirected and weighted. An edge is created between two users, e.g., *u* and *u′*, when user *u* retweets or mentions a tweet generated from user *u′* or the other way around. The weight of the edge is the number of retweets or mentions between two end users. We first eliminated all edges with weights less than two to exclude the occasional interactions. Then we removed the vertices with degrees smaller than 2 to ensure active users possess frequent interactions with multiple users. In the end, we retained in total 54,381 active users.

As Twitter includes accounts of organizations as well as bots, we make use of existing methods/tools to identify these two types of accounts. We detected about 6764 organization accounts which are about 12.44% of the selected active users with the methods proposed in [7]. We use Botometer [20] to detect bot accounts. Due to the limitation of the maximum number of daily accounts that can be processed, we randomly select 10,000 users and only 1.41% are classified as bots. Considering the small number, in spite of the claimed high accuracy of Botometer, we do not remove these suspicious bot accounts.

#### 2.1.2 Vaccine-Related Tweet Streaming

With the set of active users in the targeted countries, in this step, we crawled the tweets originated or retweeted by these users that are related to COVID-19 vaccines or vaccination. Same as previous studies [8], we use keywords related to COVID-19 vaccines to filter tweets. Two methods have been adopted to select vaccine-related keywords in the literature [3]. One is called snowball sampling which iteratively enriches the initial set of keywords according to the newly downloaded messages. The other method directly constructs the set of keywords based on expert knowledge and contexts. As many keyword lists are publicly available and produce rather good coverage [9], we decided to refer to them and only selected the ones with the best coverage to keep the list short. As the tweets originated from our targeted countries are written in multiple languages, which are different from those studied in the previous works, we translated the selected keywords when necessary. After multiple rounds of manual validation, we used all the keywords containing the following words as substrings: ‘*vax*’, ‘*vaccin*’, ‘*covidvic*’, ‘*impfstoff* ’, ‘*vacin*’, ‘*vacuna*’, ‘*impfung*’. ‘*sputnikv*’, ‘*astrazeneca*’, ‘*sinovac*’, ‘*pfizer* ’, ‘*moderna*’, ‘*janssen*’, ‘*johnson*’ and ‘*biontech*’. We used the Twitter Academic Research API to search relevant tweets based on the active users’ IDs and the keywords. The API allows for downloading at most 500 tweets for each downloading request. In order to ensure a good coverage, we constructed a request for each user every month. This enables us to obtain an acceptable coverage due to the small likelihood that a user posts more than 500 tweets related to COVID-19 vaccines in one month. In total, we downloaded 2,198,090 tweets whose IDs are released.

## 3 Data Annotation

### 3.1 Annotator Training and Consolidation

Since the number of downloaded tweets exceeds our capacity to annotate, we selected a number of tweets that can well represent the linguistic features of COVID-19 vaccination related tweets. Specifically, we first sorted the downloaded tweets in the descending order by their numbers of times being retweeted. We then removed the most frequently retweeted tweet and added it to the list of tweets to annotate iteratively until every active user has at least one posted or retweeted message in the list. In total, we selected 17,934 tweets.

We hired 10 bachelor students to manually annotate the sampled tweets. All annotators are proficient in at least two of the four official languages of the targeted countries, and, in the meantime, were active on Twitter. One author of this paper acted as the coordinator in charge of annotator training and annotation consolidation. Each annotator received a tutorial from the coordinator explaining the semantics of all labels with examples. We also distributed a guideline illustrating the workflow on the Doccano platform^3^ we built to collect annotators’ input. To ensure that all annotators held the correct understanding, we conducted a pilot annotation process in which all annotators were first asked to annotate 100 tweets. The coordinator verified their annotations and provided additional explanations if necessary. We repeated the process with another 100 tweets. After two rounds of training, annotators succeeded in understanding the labels and also became familiar with the Doccano platform.

We first selected one annotator to annotate all the tweets and this full annotation took approximately 60 h. We then randomly assigned to each of the rest 9 annotators around 2000 tweets and asked them to validate the labels. Meanwhile, the coordinator went through all annotated tweets. When an annotator disagrees with the labels given by the first annotator, he/she adds new labels according to his/her own understanding. This validation took the coordinator about 60 h and each of the other annotators 4 h. In this way, our annotation strategy ensured each tweet will be labeled three times. To solve the conflicts, the coordinator consolidated all annotations. The label agreed by at least two annotators is set as the final annotation. For those with three different labels, the coordinator communicated with the other two annotators and picked the most appropriate one.

### 3.2 Annotator Agreement

To ensure the quality of our annotations, we leverage three widely accepted measurements to quantitatively evaluate the inter-annotator reliability for each label: Average Observed Agreement (AOA) [10], Fleiss’ kappa [10], and Krippendorff's Alpha [11]. AOA is the average observed agreement between any pair of annotators. The term “observed agreement” in AOA refers to the proportion of labels two annotators agree with. Both Fleiss’ kappa and Krippendorff's Alpha are applicable to measure the agreement between a fixed number of annotators, with the difference that Krippendorff's Alpha can handle missing labels. The values of all the three measurements range from 0 to 1, where 0 indicates complete disagreement and 1 indicates absolute agreement. For Fleiss’ Kappa, 0.41–0.60, 0.61–0.80, and 0.81–1.0 are considered as moderate agreement, substantial agreement, and excellent agreement, respectively [10]. Krippendorff's Alpha is more rigorous than normal standards [11]. Values between 0.667 and 0.800 are deemed acceptable, while values greater than or equal to 0.8 are considered highly reliable [11].

Table 2 summarizes the inter-annotator agreement for each annotation label. We can see that for all labels, AOA scores range from 0.61 to 0.83. This implies that most of the annotations have at least two annotators in agreement. The values of the other two measurements are close. The annotators achieved the highest rank of agreement according to Fleiss’ kappa and Krippendorff's Alpha for both the labels NE and OT and the second highest rank on labels PO and NE. The annotators’ agreement on PD falls drastically compared to other labels, but still remains moderate according to the ranking criteria of the Fleiss’ Kappa measurement.

**Table 2:** Inter-annotator agreement (PO: Positive, NG: Negative, NE: Neutral, PD: Positive but dissatisfaction, OT: Off-topic). The large values of all the three measurements indicate a high-level agreement among annotators.

Label	AOA	Feliss’ kappa	Krippendorf's Alpha
PO	0.72	0.73	0.73
NG	0.82	0.88	0.88
NE	0.74	0.78	0.77
PD	0.61	0.63	0.62
OT	0.83	0.87	0.86

This can be explained by our difficulties during annotation in dealing with the special linguistic features of PD tweets, i.e., frequently used negative terms or sarcastic expressions. A closer look will lead to another observation that the extent of agreement on the label PO is slightly lower. A careful manual investigation reveals that a large proportion of disagreed annotations also attribute to the sarcasm and irony made to express their opinions about anti-vaccination. This identified challenge to handle sarcasm is consistent with the previous finding that people frequently are confused by sarcasm, which makes comprehension difficult [12]. We take the following tweet as an example: I am very disappointed! 16 days after my first injection of the vaccine against #COVID19 I still do not get the 5 g. This tweet uses ironic expressions joking about anti-vaccination comments, but in fact delivers a definite supporting attitude for vaccination. Such tweets produced misunderstandings among annotators, which are solved in our consolidation phase.

## 4 Vaccination Attitude Calculation with NLP

The application of deep learning and machine learning has revolutionized NLP, especially in extracting opinions or sentiments from textual contents. Compared to machine learning models which rely on manually constructed features, deep learning models can learn effective features automatically with little manual intervention. Extensive empirical evidence has proved the overwhelming performance of deep learning models in NLP studies [13]. We trained and ran several well-established NLP models based on machine learning and deep learning with our annotated tweets. A good performance of the trained models in classifying tweets will attest the utility and trustworthiness of our annotation.

*Experiment setup***.** We select Random Forest (RF) and Support Vector Machines (SVM) as the representative machine learning models due to their wide use. Regarding deep learning models, we use BERT [14], RoBERTa [15], and DistilBERT [16], CamemBERT [17] and GottBERT [18]. Note that CamemBERT and GottBERT are only applicable for single languages. Specifically, we apply CamenBERT on French tweets and GottBERT for German tweets. Such deep learning models are pretrained and produce a low-dimensional representation for any given piece of text, which can be used as input for downstream classification methods.

We preprocess the tweets by removing mentions of other users with ‘@’, quoted hyperlinks and ‘RT’ which stands for “retweet”. We remove tweets with the label ‘off-topic’ due to their small proportion. To test the models’ capability in dealing with multilingualism, we construct three datasets: the original annotated tweets, the French-language annotated tweets and the German-language annotated tweets. We divide each of these three datasets into training, testing and validation set with the ratio 80%, 10% and 10%, respectively. To train the selected machine learning models, we use TfidfVectorizer to convert the preprocessed tweets into the bag of n-gram vectors. We use grid search as the optimizer for SVM and RF. For deep learning methods, we adopt their publicly available implementation for text embedding and keep their default settings. The text embeddings are then sent to a fully-connected ReLU layer with dropout. A linear layer is added on the top of the final outputs for regression with softmax as the activation function. We use CrossEntropyLoss as the loss function and Adam as the optimizer. All models are trained for 30 epochs for optimization with the learning rate of 0.00001, and batch size of 32. We set the maximum sequence length as 128, which defines the maximum number of tokens in a tweet that can be processed.

*Result analysis***.**Table 3 shows the classification performance evaluated with conventional measurements. First, we can see that the deep learning methods outperform the machine learning methods in classifying both multilingual tweets and those in single languages, and their performances are close. This confirms the findings in [8]. Second, the results show that multilingualism affects the classification performance of deep learning models, although we use a pre-trained model specifically for classifying tweets in multiple languages. Third, when trained on a single language, multilingual models can produce comparable performance to the models specifically designed for single languages.

**Table 3:** Classification results for different benchmarks. The first five models are applicable for multilingual texts while the last two can only work on monolingual texts.

	Multilingual				French				German			
Model	Precision	Recall	F1	Accuracy	Precision	Recall	F1	Accuracy	Precision	Recall	F1	Accuracy
RF	0.4317	0.3219	0.4471	0.4749	0.5676	0.4829	0.4754	0.5510	0.5148	0.4978	0.4611	0.4503
SVM	0.4001	0.3816	0.4380	0.4263	0.5004	0.4256	0.4141	0.4998	0.4954	0.4037	0.4093	0.4719
mBERT	0.6622	0.5769	0.6132	0.6466	0.7016	0.6933	0.7004	0.7184	0.6999	0.6875	0.6919	0.7038
xlm-roberta	0.6801	0.5848	0.6271	0.6618	0.7023	0.7018	0.7145	0.7086	0.7102	0.6971	0.7081	0.7079
distill-mbert	0.6768	0.5834	0.6287	0.6601	0.6978	0.6916	0.7084	0.7065	0.7094	0.7004	0.7068	0.7071
CamenBERT	–	–	–	–	0.7147	0.7136	0.7222	0.7120	–	–	–	–
GottBERT	–	–	–	–	–	–	–	–	0.7165	0.7046	0.7199	0.7136

By comparing with the models’ performance on other classifying tasks in the literature [19], we observe that the models can achieve the same-level performances. This implies that our annotation is trustworthy and useful for future research on vaccination attitude learning. The results we showed in Table 3 can thus be referred to as benchmarks for comparison.

## Ethics Statements

We strictly adhered to the Twitter Developer Agreement and Policies3 in the collection and distribution of data. Our release is also compliant with the EU General Data Protection Regulation (GDPR). The released data do not contain any personally identification information. Only tweet IDs and annotations are published.

## CRediT authorship contribution statement

**Ninghan Chen:** Data curation, Visualization, Investigation. **Xihui Chen:** Conceptualization, Methodology, Writing – original draft. **Jun Pang:** Supervision, Validation, Writing – review & editing.

## Declaration of Competing Interest

The authors declare that they have no known competing financial interests or personal relationships that could have appeared to influence the work reported in this paper.

## Data Availability

A multilingual dataset of COVID-19 vaccination attitudes on Twitter (Original data) (Zenodo).

## Footnotes

1 https://www.nltk.org/api/nltk.html

2 https://www.pfizer.com/news/press-release/press-release-detail/pfizer-and-biontech-announce-vaccine-candidate-against

3 https://github.com/doccano/doccano
